# Neonatal Genetic Delivery of Anti-Respiratory Syncytial Virus (RSV) Antibody by Non-Human Primate-Based Adenoviral Vector to Provide Protection against RSV

**DOI:** 10.3390/vaccines7010003

**Published:** 2018-12-29

**Authors:** Rika Gomi, Anurag Sharma, Wenzhu Wu, Stefan Worgall

**Affiliations:** 1 Department of Pediatrics, Weill Cornell Medicine, New York, NY 10065, USA; rig2015@med.cornell.edu (R.G.); anurag1077@gmail.com (A.S.); wew2005@med.cornell.edu (W.W.); 2 Department of Genetic Medicine, Weill Cornell Medicine, New York, NY 10065, USA

**Keywords:** respiratory syncytial virus (RSV), immunoprophylaxis, palivizumab, chimpanzee adenovirus type 7 (AdC7), neonate, passive immunization, genetic gene delivery

## Abstract

Respiratory syncytial virus (RSV) is one of the leading causes of lower respiratory tract infection in infants. Immunoprophylaxis with the anti-RSV monoclonal antibody, palivizumab, reduces the risk for RSV-related hospitalizations, but its use is restricted to high-risk infants due to the high costs. In this study, we investigated if genetic delivery of anti-RSV antibody to neonatal mice by chimpanzee adenovirus type 7 expressing the murine form of palivizumab (AdC7αRSV) can provide protection against RSV. Intranasal and intramuscular administration of AdC7αRSV to adult mice resulted in similar levels of anti-RSV IgG in the serum. However, only intranasal administration resulted in detectable levels of anti-RSV IgG in the bronchoalveolar lavage fluid. Intranasal administration of AdC7αRSV provided protection against subsequent RSV challenge. Expression of the anti-RSV antibody was prolonged following intranasal administration of AdC7αRSV to neonatal mice. Protection against RSV was confirmed at 6 weeks of age. These data suggest that neonatal genetic delivery of anti-RSV antibody by AdC7αRSV can provide protection against RSV.

## 1. Introduction

Respiratory syncytial virus (RSV) is one of the leading causes of lower respiratory tract infections in children, with a high morbidity and mortality, especially in developing countries [1,2,3]. Most children are infected during their first year of life, and infants under six months of age are at highest risk for severe disease, especially those with bronchopulmonary dysplasia or cyanotic heart disease [4,5]. 

Despite the extensive efforts over decades, there are no effective vaccines or treatments for RSV. Immunoprophylaxis with the neutralizing monoclonal antibody, palivizumab, is the only available drug to prevent infections with RSV [6,7,8]. Although palivizumab is safe and well-tolerated for prophylactic use, its use is limited to high-risk infants due to the high costs and the need for monthly intramuscular injections during the RSV season [9]. Since most deaths attributed to RSV occur in low-income countries where expensive prophylaxis is not affordable [3], more cost-effective strategies are needed to spare more infants at risk from severe RSV disease.

Genetic delivery of neutralizing antibodies using gene transfer vectors is an alternative strategy to achieve sustained expression of neutralizing antibodies. Among all currently available viral vectors, adenovirus (Ad) is one of the most efficient gene delivery systems, but pre-existing immunity against common human serotypes, such as Ad5, is hampering its use in humans. Non-human primate-derived Ad, such as chimpanzee Ad serotype 7 (AdC7), are alternative vectors and are less likely to be affected by pre-existing immunity [10,11,12]. We have previously reported that anti-Ad5 neutralizing antibodies do not cross-neutralize AdC7, and that maternal immunization of mice with the AdC7 vector expressing RSV vaccine antigen can protect their pups against RSV infection [13]. Therefore, we adopted AdC7 as a gene transfer vector to deliver anti-RSV neutralizing antibody to infants, expecting a successful application in the presence of maternal anti-human Ad antibodies.

Anti-Ad immunity induced by the vector limits long-term expression of the transgene and is thus another limitation of Ad vectors. However, immune tolerance may allow longer persistence when the vector is administered to neonates. The neonatal immune system is much less likely to develop a vigorous immune response to transgenic proteins [14,15]. In this study, we administered AdC7 vector expressing anti-RSV antibody (AdC7αRSV) to neonatal mice and evaluated the efficacy of the delivered anti-RSV antibody to protect against RSV infection.

## 2. Materials and Methods 

### 2.1. Mice

BALB/c mice were purchased from The Jackson Laboratories (Bar Harbor, ME, USA) housed under specific pathogen-free conditions, and bred to obtain neonatal mice. Adult female BALB/c mice were used at 8 weeks of age, and neonatal mice were used between 24 and 48 h after the birth. All animal studies were conducted in accordance with the protocols reviewed and approved by the Weill Cornell Institutional Animal Care and Use Committee (protocol number 2015-0011). All efforts were made to minimize the suffering of the animals.

### 2.2. RSV

The RSV strains A2 (VR-1540: ATCC), Line19 and rA2-F19 (kindly provided by ML Moore, Emory University, Atlanta, GA, USA) were propagated in Hep-2 cells, purified and quantified as described previously [16].

### 2.3. Generation of an AdC7 Vector Expressing Murine Anti-RSV IgG (AdC7αRSV)

The recombinant Ad vectors used in this study are replication defective *E1*-, *E3*-deleted Ad vectors based on the chimpanzee AdC7. The AdC7 plasmid pPan-GFP (kindly provided by JM Wilson, University of Pennsylvania, Philadelphia, PA, USA) was digested with I-Ceul and PI-Scel, and the expression cassette of anti-RSV antibody carrying (5′ to 3′) the cytomegalovirus promoter/enhancer followed by cDNAs encoding the anti-RSV light chain, the poliovirus internal ribosomal entry site (IRES), the anti-RSV heavy chain, and the SV40 polyadenylation signal [17] was inserted into the *E1* region using the same restriction enzyme sites. AdC7GFP, an AdC7 vector with the green fluorescent protein cDNA under control of a prokaryotic promoter which does not lead to transgene expression in mammalian cells, were used as controls. The AdC7αRSV vectors were propagated in HEK-293 cells and purified by centrifugation twice through a CsCl gradient as previously described [18], and the particle units (pu) were determined spectrophotometrically [19]. 

### 2.4. Western Blot Analysis

To confirm the expression of anti-RSV IgG in vitro, supernatants of A549 cells infected with AdC7αRSV were separated by SDS-PAGE under both non-reducing and reducing conditions. Following transfer to a polyvinylidene difluoride (PVDF) membrane (Bio-Rad Laboratories, Hercules, CA, USA) murine IgG was detected using a horseradish peroxidase (HRP)-conjugated sheep anti-mouse IgG antibody (Sigma, St. Louis, MO, USA) and Immobilon Western Chemiluminescent HRP substrate (EMD Millipore, Burlington, MA, USA). The supernatant of mock-infected cells was used as negative control. Mouse serum obtained 8 weeks following RSV infection was used as a positive control.

### 2.5. Dot Blot

To evaluate binding of anti-RSV IgG to RSV, RSV Line19 (1.2 × 10^4^ pfu/spot) was blotted to the PVDF membrane (Bio-Rad Laboratories, Hercules, CA, USA) and then developed with culture supernatant of HEK-293 cells infected with AdC7αRSV followed by the sheep anti-mouse IgG-peroxidase antibody as described above. Ad5 (2.0 × 10^6^ pu/spot) was blotted as control.

### 2.6. Plaque Reduction Assay

Serial dilutions of supernatants from HEK-293 cells that had been infected for 48 h with AdC7αRSV were incubated with RSV Line19 (5 × 10^3^ pfu/mL) for 1 h at 37 °C, and then added to Vero cells. The number of plaques was quantified after 4 days as previously described [16].

### 2.7. Expression of anti-RSV IgG In Vivo

AdC7αRSV or AdC7GFP (5 × 10^10^ pu each) diluted in 40 μl PBS were administered intranasally or intramuscularly to 8-week-old female BALB/c mice. Neonatal mice received either 3 × 10^10^ pu (5μL) or 6 × 10^10^ pu (10 μL) of the vectors intranasally. The levels and kinetics of anti-RSV IgG following administration of AdC7αRSV were quantified in serum and bronchoalveolar lavage (BAL) by ELISA. Serial dilutions of serum and BAL were added to flat-bottomed 96-well EIA/RIA plates (Corning, Corning, NY, USA) coated with 1μg/mL of human anti-palivizumab clone AbD23967 (HCA261, Bio-Rad-Antibodies, Hercules, CA, USA), followed by PBST + 5% blotting grade blocker (Bio-Rad Laboratories, Hercules, CA, USA. Detection was performed using an HRP-conjugated sheep anti-mouse IgG (Sigma, St. Louis, MO, USA) in PBS + 1% blotting grade blocker and substrate (hydrogen peroxide/tetramethylbenzidine) (R&D systems, Minneapolis, MN, USA) and the absorbance at 450 nm was measured. Titers were calculated with a log (OD)–log(dilution) interpolation model, with detection cut-off equal to 2-fold the background absorbance. Half-life (*t*_1/2_) was calculated by the formula:

*t*_1/2_ = *t* ∗ ln(2)/ln(*N*_0_/*N*_t_), (1)

where *t* = time elapsed, *N*_0_ = titer at 1 week, and *N*_t_ = titer at 4 weeks after the administration of AdC7αRSV.

### 2.8. Protection Against RSV Infection Following AdC7αRSV Administration

Mice were challenged 3 days or 6 weeks following Ad vector administration with RSV Line19 or RSV A2 (each 1 × 10^6^ pfu) via the intranasal route. Four days after the challenge, RSV was quantified in lung homogenates by plaque assay, or viral RNA was quantified by real-time PCR as described previously [13]. For the detection of RSV Line19 viral RNA, Line19 NS1-specific primers (Forward: 5′-CAGCGCTACAAAATGGAGGTTA-3′, Reverse: 5′-TTAGACCATTAGGTTGAGAGCAATGT-3′) and FAM-labeled TaqMan MGB probes (5′-ATATGGGAAATGATGGAATT-3′) were used.

### 2.9. Statistics

Statistical analyses were performed using one-way ANOVA, followed by two-tailed unpaired Student’s t-tests. Statistical significance was determined at *p* < 0.05.

## 3. Results

### 3.1. Expression of Murine Anti-RSV In Vitro

**Figure 1:**
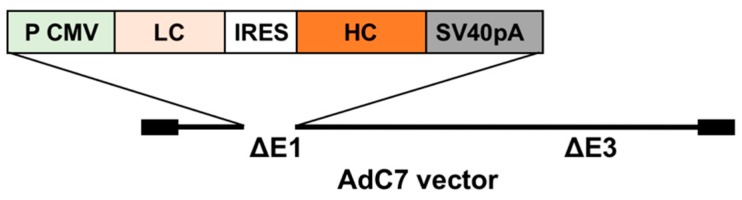
Schema of the chimpanzee adenovirus type 7 vector expressing murine anti-respiratory syncytial virus antibody (AdC7αRSV). The *E1/E3* genes of AdC7 are deleted (ΔE1/ΔE3) and replaced by the expression cassette of the anti-RSV antibody cDNAs using the restriction enzyme sites I-CeuI and PI-SceI. The expression cassette includes the cytomegalovirus promoter (P CMV), followed by cDNAs encoding the anti-RSV light chain (LC), the poliovirus internal ribosome entry site (IRES), the anti-RSV heavy chain (HC), and SV40 polyadenylation signal (SV40 pA).

AdC7αRSV (Figure 1) was generated and propagated in HEK-293 cells. 

**Figure 2:**
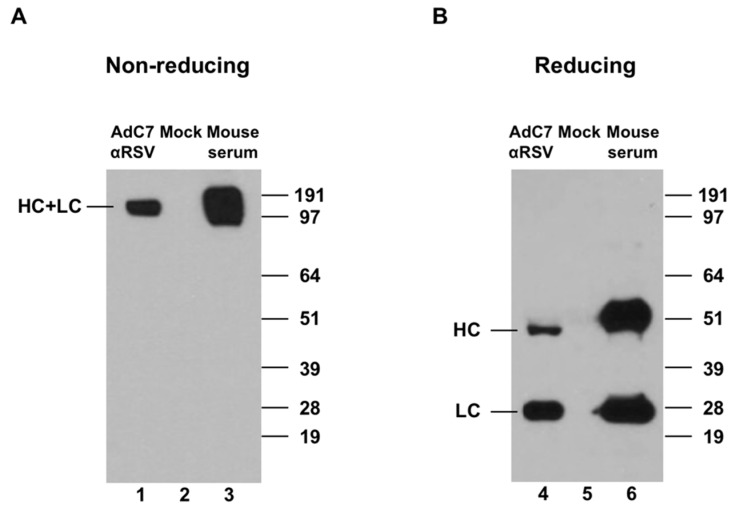
Expression of murine anti-respiratory syncytial virus (anti-RSV) IgG in vitro. Anti-RSV IgG in supernatants of A549 cells infected with chimpanzee adenovirus type 7 vector expressing murine anti-RSV IgG (AdC7αRSV) was detected by Western Blot analysis. (**A**) Expression of the full-length murine IgG under non-reducing conditions. (**B**) Expression of the heavy chain (HC) and light chain (LC) of murine IgG under reducing conditions. The supernatant of mock-infected cells was used as a negative control (lanes 2, 5). Mouse serum 8 weeks post infection with RSV was used as a positive control (lanes 3, 6). Detection was with a horseradish peroxidase (HRP)-conjugated sheep anti-mouse IgG and HRP chemiluminescence substrate.

To confirm expression of murine anti-RSV IgG in vitro, A549 cells were infected with purified AdC7αRSV, and cell culture supernatants were assessed by Western Blot analysis (Figure 2). Under non-reducing conditions, a complex of 150 kDa, corresponding to the size of the completely assembled murine IgG, was detected (Figure 2A, lane 1). Under reducing conditions, individual heavy chains (HC, 50 kDa) and light chains (LC, 25 kDa) were detected (Figure 2B; lane 4). 

**Figure 3:**
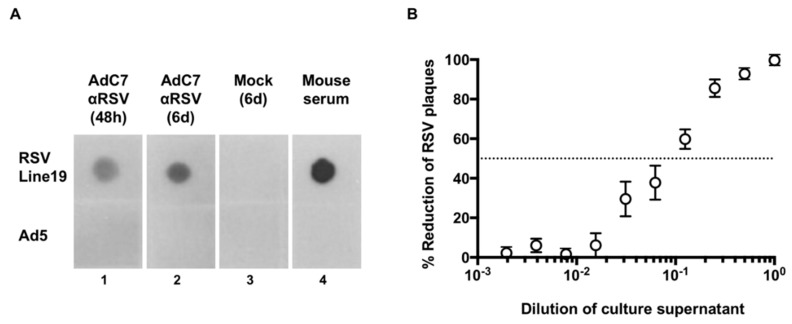
Assessment of anti-respiratory syncytial virus (anti-RSV) IgG expression in vitro. Supernatants of HEK-293 cells infected with AdC7αRSV were assessed for the presence of functional anti-RSV IgG. (**A**) Binding to RSV. Supernatants were incubated with RSV Line19 or Ad5 (control) immobilized on a polyvinylidene difluoride (PVDF) membrane followed by an HRP-conjugated sheep anti-mouse IgG. Mouse serum 8 weeks post infection with RSV was used as a positive control (lane 4). (**B**) Plaque-reduction assay. Serial dilutions of supernatants were incubated with RSV Line19 (5 × 10^3^ pfu/mL) for 1 h, followed by infection of Vero cells. Data are shown as % reduction of plaques after 4 days, with mean ± SEM of 4 replicates.

The binding ability of the expressed anti-RSV IgG to RSV was confirmed by dot blot ELISA using RSV Line19 binding to cell culture supernatants of HEK-293 cells infected with AdC7αRSV (Figure 3A, lanes 1, 2). Plaque-reduction assay showed dose-dependent neutralization of RSV by the antibodies (Figure 3B).

### 3.2. Assessment of Anti-RSV IgG Delivered by AdC7αRSV In Vivo

**Figure 4:**
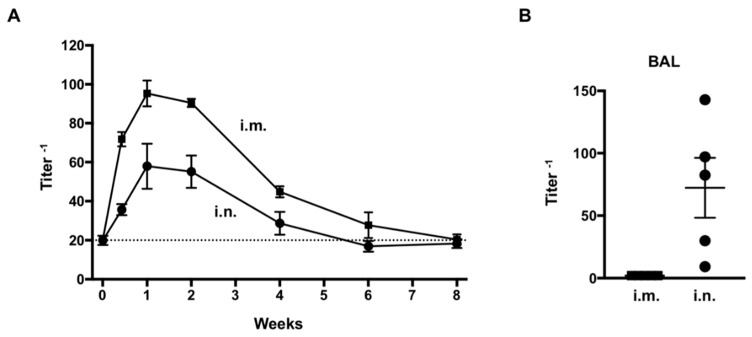
Assessment of anti-respiratory syncytial virus (anti-RSV) IgG expression in adult mice. (**A**) Kinetics of anti-RSV IgG in the serum following intranasal (i.n.) or intramuscular (i.m.) administration of AdC7αRSV (5 × 10^10^ pu). Titers were measured by ELISA. Data are shown as mean ± SEM of 4 mice per group. (**B**) Anti-RSV IgG in the bronchoalveolar lavage (BAL) 1 week following administration of AdC7αRSV (5 × 10^10^ pu). Titers were measured by ELISA. Data are shown with mean ± SEM.

The kinetics of anti-RSV IgG in the serum of adult mice following intranasal or intramuscular administration of AdC7αRSV showed that the anti-RSV IgG titer peaked at one week following administration, and then gradually decreased to non-specific response levels by eight weeks (Figure 4A). The estimated half-life was 19 days. Although the titers were higher following intramuscular administration, the kinetics of anti-RSV levels over time were similar between the two routes of administration. In contrast, anti-RSV IgG in the BAL at one week was only detectable following intranasal administration (Figure 4B).

### 3.3. Protection Against RSV Infection Following AdC7αRSV Administration in Adult Mice

**Figure 5:**
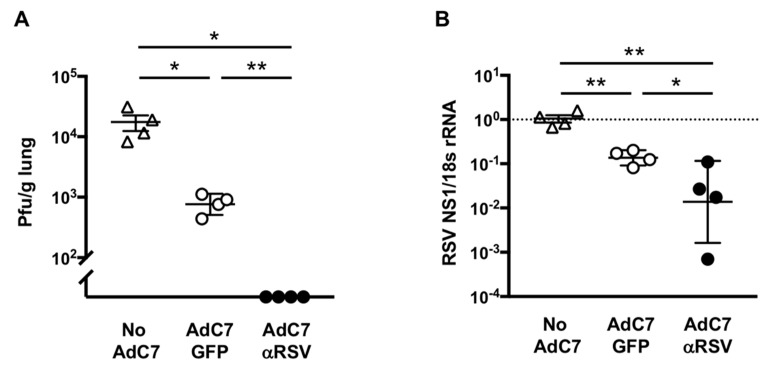
Protection against RSV infection following AdC7αRSV administration to adult mice. AdC7αRSV, AdC7GFP (5 × 10^10^ pu) or PBS (No AdC7 control) were intranasally administered to 8-week-old BALB/c mice, followed by RSV Line19 (10^6^ pfu) challenge 3 days later. (**A**) RSV viral loads in the lungs 4 days after the RSV challenge by plaque assay. (**B**) RSV genome expression in the lungs 4 days after the challenge by RT-qPCR. Data are shown with mean ± SEM. * and ** denote *p* < 0.05 and *p* < 0.01, respectively.

To evaluate if AdC7αRSV can deliver sufficient antibody to protect against RSV infection, AdC7αRSV, AdC7GFP or PBS (No AdC7 control) were intranasally administered to eight-week-old female BALB/c mice, followed by RSV Line19 infection three days later. RSV viral loads in the lungs of mice that had received AdC7αRSV were lower (below detection level) compared to mice that had received AdC7GFP (Figure 5A; *p* < 0.01). Likewise, RSV viral genomes also decreased in mice that had received AdC7αRSV compared to AdC7GFP (Figure 5B; *p* < 0.05). This suggests that intranasal administration of AdC7αRSV provides protection against RSV infection in adult mice.

### 3.4. Protection Against RSV Following AdC7αRSV Administration to Neonatal Mice

**Figure 6:**
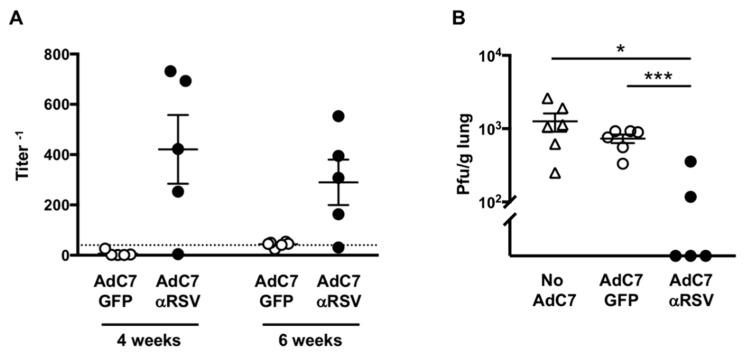
Protection against RSV infection following AdC7αRSV administration to neonatal mice. AdC7αRSV or AdC7GFP (6 × 10^10^ pu) were intranasally administered to 1-day-old BALB/c mice, followed by RSV A2 (10^6^ pfu) challenge at 6 weeks of age. (**A**) Anti-RSV IgG titer in serum before the RSV challenge. Serum was collected at 4 and 6 weeks, and titers were measured by ELISA. Data are shown with mean ± SEM. (**B**) RSV viral loads in the lungs 4 days after the RSV challenge by plaque assay. Data are shown with mean ± SEM. * and *** denote *p* < 0.05 and *p* < 0.005, respectively.

To evaluate if AdC7αRSV administered to neonatal mice can lead to protection against RSV, AdC7αRSV or AdC7GFP were intranasally administered to one-day-old mice. Anti-RSV IgG was detected in 4 out of 5 mice that had received AdC7αRSV at four and six weeks of age (Figure 6A). The kinetics of anti-RSV IgG in the serum of neonatal mice after four weeks of age following intranasal administration of AdC7αRSV showed that the titers were the highest at four weeks and then gradually decreased, but stayed at detectable levels until nine weeks (Figure S1A). Serum anti-RSV IgG titers were also at detectable levels 14 weeks following intranasal administration of AdC7αRSV. The mice that had received a higher dose of AdC7αRSV tended to have higher anti-RSV IgG titers, particularly in lung homogenate supernatants (Figure S1B). Importantly, challenge with RSV at six weeks of age showed a reduction of viral loads only in the mice that had received AdC7αRSV, with undetectable RSV level in three out of five mice, compared with the mice that had received AdC7GFP or that had not received any Ad vector (Figure 6B). Protection against RSV was also evaluated at a later time point; Anti-RSV IgG expression was confirmed in the mice that had received AdC7αRSV at four and eight weeks of age (Figure S2A). Challenge with RSV at 10 weeks of age also showed a reduction of viral loads in three out of four mice that had received AdC7αRSV, compared with the mice that had received AdC7GFP or that had not received any Ad vector (Figure S2B). These data suggest that administration of AdC7αRSV to neonatal mice can lead to protection against RSV, and prolonged protective immunity compared to adult mice can be provided.

## 4. Discussion

In this study, we showed that intranasal administration of AdC7αRSV could provide protection against RSV infection. The administration route of Ad vector may greatly contribute to the efficiency of local delivery to the airway. Intravenous administration of palivizumab was shown to be effective in rodents and humans, effectively reducing lung RSV load in the lung of cotton rats [20] and being detectable in human nasal washes [21]. It is known that intramuscularly injected palivizumab is slowly absorbed and maximum serum concentrations are reached at three to five days [22], but the kinetics of lung and airway levels are less well-known. Our data showed that the anti-RSV IgG was detected in the BAL after intranasal administration but not intramuscular administration at the time of peak serum IgG levels (at one week). The intranasal administration route may be advantageous through the production of the neutralizing antibodies directly by the respiratory mucosal cells, the primary target of RSV. Future studies should examine the cellular source of the antibody within the respiratory mucosa, and include more analyses of the nose-associated lymphoid tissue (NALT) immune cells.

Immune responses in the respiratory tract can be altered by prior viral infections [23,24]. The recent study [25] revealed that nasal priming by viruses can influence lung immunity and can induce protective immunity against heterologous viral infection. Our data in the adult mice showed some protection against RSV also in the mice that had received the control AdC7GFP vector (Figure 5A). Although the protection against subsequent RSV infection was much greater in the mice that had received AdC7αRSV, the AdC7 vector itself seemed to have a priming effect to the lung immunity.

Neonatal adaptive immune responses show a great deal of variability, ranging from non-responsiveness to fully mature function. It has been shown that newborn mice can be tolerized to Ad vectors, and that repeat administration of Ad vector could be possible [26]. Thus, immune tolerance can be expected when vectors are administered to neonates. Administration of AdC7αRSV to one-day-old mice resulted in prolonged antibody expression compared to adult mice. Another potential advantage of neonatal gene delivery is the higher vector particles-to-cell ratio, requiring a lower relative dose [15]. In addition, Ad vector delivery seems to be more efficient in the neonatal lung compared to adult lungs [27]. Our data showed that the serum anti-RSV IgG titers are higher after neonatal administration compared to adult administration, albeit with the same dose.

To reduce the burden of disease caused by RSV, there is a strong consensus that focus should be placed on children in their first six months of life when the risk of severe RSV-associated respiratory disease is highest [8]. Immunization to provide the protective immunity lasting throughout the vulnerable period would be an ideal strategy to protect children from RSV infection. We focused on passive immunization in this study, but the combination of AdC7-based passive immunization and AdC7-based active immunization can be an attractive strategy to provide complete protective immunity throughout the vulnerable period. Since mature responses to vaccines can also be expected in the neonatal immune system [14], simultaneous administration of AdC7 which carries anti-RSV neutralizing antibody and RSV vaccine antigen may provide both passive and active immunization. An appropriate vaccine antigen that is not overlapped with the antigenic site for palivizumab would be required to make this strategy successful.

We did not include wild-type RSV or other RSV vaccine controls in this study. Since our primary interest was to determine if genetic delivery of anti-RSV antibody by AdC7 vector can be effective in neonatal mice, we used AdC7 control vector without transgene expression (AdC7GFP). Future studies should include wild-type RSV or vaccine controls to further evaluate the effectiveness of this vector strategy, even if wild-type RSV is not a realistic vaccine alternative for neonates.

The half-life of the delivered antibody in our study was 19 days, which is compatible with a known half-life of palivizumab. Prolonged expression was seen when the vectors were administered to neonatal mice, but we could not estimate the half-life of antibodies since a serial collection of neonatal serum was not feasible. Several explanations can be possible for the prolonged expression. The antibody may have had a longer half-life than in adult mice because of the unique immunological properties of neonatal mice. The efficiency of gene delivery may have been greater due to the higher susceptibility to Ad of neonatal lungs. Recently, the development of new anti-RSV monoclonal antibodies went toward prolongation of the serum half-life. The amino acid modification made it possible to extend the serum half-life of the antibody [28,29]. Modifying the expression cassette on the AdC7 to deliver more efficient anti-RSV monoclonal antibody with extended half-life itself may enable further long-term protection against RSV.

## 5. Conclusions

In summary, our data showed that intranasal administration of AdC7αRSV to neonatal mice provided prolonged expression of anti-RSV antibody to protect against RSV infection. Neonatal immune responses have been mainly studied in mice, but there are some indications that a similar situation is true in humans in early life [14]. We demonstrated the potential of neonatal genetic delivery by non-human primate-based Ad vector that could efficiently deliver anti-RSV neutralizing antibody to neonatal lungs and could provide protection against RSV.

## Supplementary Materials

The following are available online at http://www.mdpi.com/2076-393X/7/1/3/s1, Figure S1: Assessment of anti-RSV IgG expression in neonatal mice, Figure S2: Protection against RSV 10 weeks following AdC7αRSV administration to neonatal mice.
