# Supplementary material for: Neonatal Genetic Delivery of Anti-Respiratory Syncytial Virus (RSV) Antibody by Non-Human Primate-Based Adenoviral Vector to Provide Protection against RSV
Source: Vaccines (Basel). 2018 Dec 29;7(1):3. doi: 10.3390/vaccines7010003 (PMC6466083; doi:10.3390/vaccines7010003)
Supplement: Supplementary file 1 [file vaccines-07-00003-s001.pdf]

vaccines

MDPI

Supplementary Materials: Neonatal Genetic Delivery of Anti-Respiratory Syncytial Virus (RSV) Antibody by Non-Human Primate-Based Adenoviral Vector to Provide Protection against RSV

Rika Gomi [1], Anurag Sharma [1], Wenzhu Wu [1] and Stefan Worgall [1,2,*]

[1] Department of Pediatrics, Weill Cornell Medicine, New York, NY, 10065 USA; rig2015@med.cornell.edu (R.G.); anurag1077@gmail.com (A.S.); wew2005@med.cornell.edu (W.W.)

[2] Department of Genetic Medicine, Weill Cornell Medicine, New York, NY 10065 USA

* Correspondence: stw2006@med.cornell.edu; Tel.: +1-646-962-6236

Received: 1 December 2018; Accepted: 22 December 2018; Published: 29 December 2018

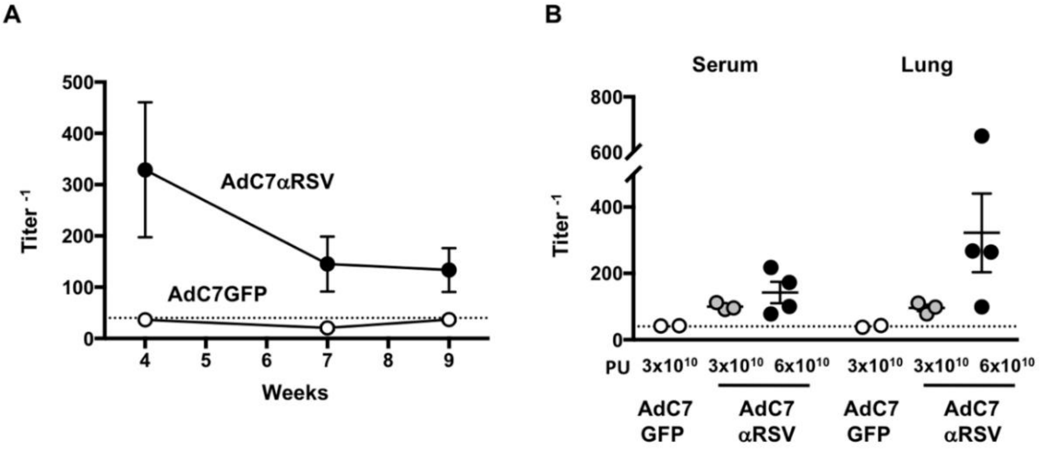

Figure S1. Assessment of anti-respiratory syncytial virus (anti-RSV) IgG expression in neonatal mice. (**A**) Kinetics of anti-RSV IgG. Chimpanzee adenovirus type 7 vector (AdC7) expressing murine anti-RSV antibody (AdC7αRSV) or AdC7 with green fluorescent protein but without transgene expression (AdC7GFP) (3×10^{10} pu each) was intranasally administered to 1-day-old BALB/c mice. Serum was collected at 4, 7 and 9 weeks after the administration and titers were measured by ELISA. The titers in mice that had received AdC7αRSV are shown as mean ± SEM of 7 mice per group. (**B**) Anti-RSV IgG in the serum and lung homogenate supernatants after 14 weeks following Ad vector administration. AdC7αRSV (3×10^{10} or 6×10^{10} pu) or AdC7GFP (3×10^{10} pu) was intranasally administered to 1-day-old BALB/c mice. Serum and Lungs were collected 14 weeks after the administration and titers were measured by ELISA. The titers in mice that had received AdC7αRSV are shown with mean ± SEM.

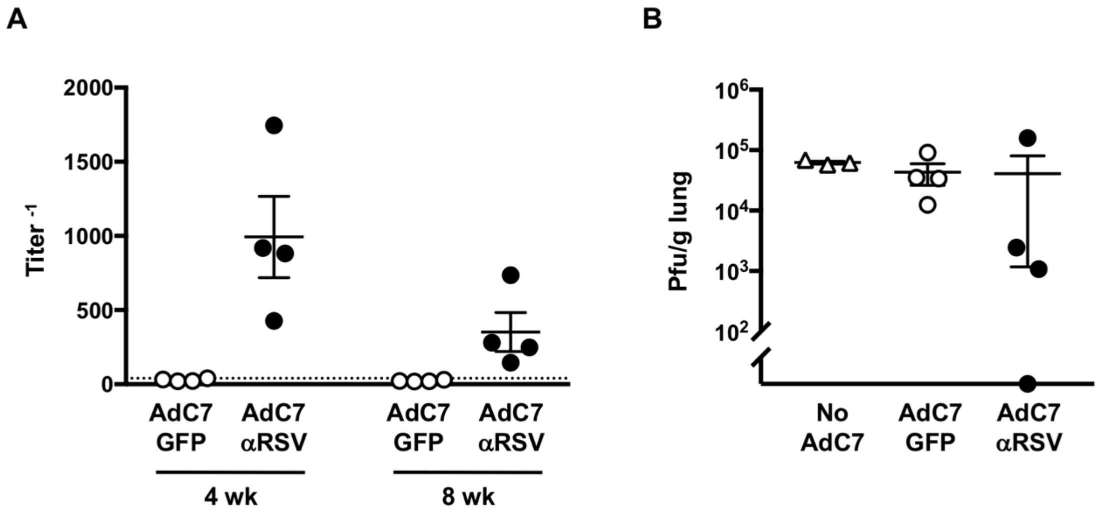

Figure S2. Protection against RSV infection 10 weeks following AdC7αRSV administration to neonatal mice. AdC7αRSV or AdC7GFP (7×10^{10} pu) were intranasally administered to 1-day-old BALB/c mice followed by RSV rA2-L19 (5×10^{6} pfu) challenge at 10 weeks of age. (**A**) Anti-RSV IgG titers in the serum before the RSV challenge. Serum was collected at 4 and 8 weeks after the administration of AdC7αRSV and titers were measured by ELISA. The titers in mice that had received AdC7αRSV are shown with mean ± SEM. (**B**) RSV viral loads in the lungs 4 days after the RSV challenge by plaque assay. Titers are shown with mean ± SEM.
